# Carbon Dioxide Improves Phosphorus Nutrition by Facilitating the Remobilization of Phosphorus From the Shoot Cell Wall in Rice (*Oryza sativa*)

**DOI:** 10.3389/fpls.2019.00665

**Published:** 2019-05-22

**Authors:** Xiao Fang Zhu, Xiao Long Zhang, Xiao Ying Dong, Ren Fang Shen

**Affiliations:** ^1^ State Key Laboratory of Soil and Sustainable Agriculture, Institute of Soil Science, Chinese Academy of Sciences, Nanjing, China; ^2^ University of Chinese Academy of Sciences, Beijing, China

**Keywords:** ethylene, phosphorus (P), cell wall, carbon dioxide (CO_2_), pectin, pectin methylesterase (PME)

## Abstract

Phosphorus (P) starvation leads to increased reutilization of cell wall P in rice (*Oryza sativa*). Carbon dioxide (CO_2_) is involved not only in plant growth and development but also in the response to abiotic stresses. However, it remains unclear whether CO_2_ affects the reutilization of cell wall P in rice when subjected to P deficiency. In the present study, elevated CO_2_ (600 μl·L^−1^) significantly increased the soluble P content in shoots when compared with ambient CO_2_ (400 μl·L^−1^). This positive effect was accompanied by an increase of pectin content, as well as an increase of pectin methylesterase (PME) activity, which results in P release from the shoot cell wall, making it available for plant growth. P deficiency significantly induced the expression of phosphate transporter genes (*OsPT2*, *OsPT6*, and *OsPT8*) and decreased the P content in the xylem sap, but elevated CO_2_ had no further effect, indicating that the increased soluble P content observed in shoots under elevated CO_2_ is attributable to the reutilization of shoot cell wall P. Elevated CO_2_ further increased the P deficiency-induced ethylene production in the shoots, and the addition of the ethylene precursor 1-amino-cyclopropane-1-carboxylic acid (ACC) mimicked this effect, while the addition of the ethylene inhibitor aminoethoxyvinylglycine (AVG) abolished this effect. These results further support the role of ethylene in the alleviation of P deficiency under elevated CO_2_. Taken together, our results indicate that the improvement of P nutrition in rice by elevated CO_2_ is mediated by increasing the shoot cell wall pectin content and PME activity, possibly *via* the ethylene signaling pathway.

## Introduction

Inorganic phosphate (Pi), the main form of phosphorus (P) taken up by plants, is essential for the normal growth of plants (Wu et al., 2013). However, due to the chemical fixation of P to cations such as Al and Fe, or to other compounds, which creates insoluble, inaccessible complexes, P deficiency is one of the major limiting factors for crop production (Vance et al., 2003; Bhadouria et al., 2017). As a consequence, large amounts of Pi fertilizers are needed (Hammond et al., 2004). As Pi-based fertilizers originate from rock phosphate and other non-renewable materials, overuse of Pi will not only exhaust the mineral P resources (Runge-Metzger, 1995; Beardsley, 2011) but also cause damage to the environment (Liang et al., 2014), such as lake eutrophication (Withers et al., 2001). Thus, breeding crops with higher yields and lower fertilization requirements is imperative for sustainable agriculture.

Plants have developed flexible strategies to cope with P limitation, such as modification of root architecture, symbiosis with mycorrhizal fungi, secretion of organic acids, modification of carbon metabolism, remobilization of Pi, and enhancing the biosynthesis of phosphate transporters (Raghothama, 1999; Smith, 2001; Shulaev et al., 2008). The cell wall also plays a key role in response to P deficiency. For example, in rice (*Oryza sativa*), the P stored in the root cell wall can be reutilized under P starvation conditions (Zhu et al., 2015). Cell walls consist of cellulose and other matrix polysaccharides such as pectin and hemicellulose (Cosgrove, 2005). Among them, only pectin has been demonstrated to participate in the reutilization of cell wall P (Zhu et al., 2015), as pectin harbors negatively charged carboxylic groups (–COO–) that have high affinity for Cd^2+^, Fe^3+^, and Al^3+^ (Blamey et al., 1990; Chang et al., 1999; Rengel and Zhang, 2003). Phosphate (PO_4_^3−^) also has high affinity for Ca^2+^, Fe^3+^, and Al^3+^, and as a result, insoluble FePO_4_, AlPO_4_, and Ca_3_(PO_4_)_2_ are formed. For instance, under aluminum stress, AlPO_4_ is the major form of P in the apoplast in buckwheat (*Fagopyrum esculentum*; Zheng et al., 2005). As the negatively charged carboxylic groups in pectin can chelate Fe^3+^, PO_4_^3−^ can be further trapped with a linkage of –COO–Fe–PO_4_. That means, ions such as Fe act as bridges connecting pectin and P; therefore, increasing the –COO– will bind Fe more tightly to the pectin and facilitate the release of pectin-trapped PO_4_^3−^ (Zhu et al., 2015). Furthermore, this pectin-mediated P reutilization is regulated by a series of phytohormones and signaling molecules, such as nitric oxide (NO; Zhu et al., 2017), abscisic acid (ABA; Zhu et al., 2018), and ethylene (Zhu et al., 2016). However, it remains unclear whether other signal molecules are involved in this cell wall P remobilization mechanism.

Carbon dioxide (CO_2_) is one of the gases responsible for global climate change (IPCC 2001). Atmospheric CO_2_ has increased more than 40% from the pre-industrial times, and current ambient CO_2_ now exceeds 400 μmol mol^−1^ (Tans and Keeling, 2018) and is expected to reach a peak of 490–1,260 μl·L^−1^ in the future (Houghton et al., 2001). The increase of CO_2_ in the atmosphere alters the growth and development of plants, which could also influence the absorption of macro- or micronutrients. Previous studies have focused on the influence of elevated CO_2_ on the plant population, community, ecosystem, and physiological scales (Gibeaut et al., 2001; Teng et al., 2006; Niu et al., 2011), while few studies have focused on the influence of elevated CO_2_ on the utilization of nutrients. CO_2_ may be involved in nutrient acquisition in plants. For example, the demand for P in Japanese red pine (*Pinus densiflora*) is related to the concentration of CO_2_ (Kogawara et al., 2006), while elevated concentrations of CO_2_ can alleviate the iron deficiency phenotype of tomato (*Solanum lycopersicum*; Jin et al., 2009). In addition, Niu et al. (2013) reported that the combination of elevated CO_2_ and nitrate can alleviate P-deficiency symptoms in *Arabidopsis thaliana* (L.) *Heynh* through alteration of morphological and physiological responses. Furthermore, Zhu et al. (2017) demonstrated that elevated CO_2_ can alleviate Al toxicity in rice by decreasing the binding capacity of the cell wall to Al. As about 49% of the total P was present in the cell wall of rice (Zhu et al., 2016), that means, the cell wall has abundant P resource for reutilization; thus the relationship among elevated CO_2_, P status, and cell walls is worth discussing and studying.

Rice is an important staple food worldwide, which accounts for approximately 21% of the caloric supply for the world’s population (Wu et al., 2011). Low P in soils significantly limits rice production (Kirk et al., 1998). Therefore, improving the utilization efficiency of P in rice is an important target for sustainable agriculture. The objective of the current study was to clarify whether elevated CO_2_ is involved in the reutilization of cell wall P in P-deficient conditions.

## Materials and Methods

### Plant Materials and Growth Conditions

Rice (*Oryza sativa) subsp. japonica* “Nipponbare” (Nip) was used in the current study. After soaking in 1% (v/v) sodium hypochlorite (NaClO) for 10 min, seeds were immediately rinsed with deionized water and then soaked in ultrapure water until germination. The germinated seeds were cultivated in calcium chloride solution (CaCl_2_; 0.5 mM) until the buds were about 1 cm long. Then, the seedlings were transferred to modified Kimura B nutrient solution for 2 weeks (Zhu et al., 2018). Seedlings of similar size were then transplanted into a 1.25-L plastic pot (4 seedlings per pot) and subjected to the ambient CO_2_ (400 μl·L^−1^) or elevated CO_2_ (600 μl·L^−1^) condition in the presence or absence of P, termed as +P, +P + CO_2_, −P, and −P + CO_2_. To investigate the crosstalk between elevated CO_2_ and ethylene, the following treatments were used: +P, +P + ACC (1-amino-cyclopropane-1-carboxylic acid), +P + AVG (aminoethoxyvinylglycine), +P + CO_2_, +P + CO_2_ + ACC, +P + CO2 + AVG, −P, −P + ACC, −P + AVG, −P + CO_2_, −P + CO_2_ + ACC, and −P + CO2 + AVG. The final concentration of ACC was 10 μM and that of AVG was 0.2 μM. The pH of the nutrient solution was 5.5.

### Measurement of the Soluble P Content

After measuring the fresh weights of roots and shoots, samples were rinsed with ultrapure water and then ground in liquid nitrogen. After extracting with 8 ml of 5 M H_2_SO_4_ for 2 h, samples were centrifuged at 12,000*g* for 8 min, and 400 μl of supernatant was transferred to a 2 ml Eppendorf tube and mixed with 200 μl of ammonium molybdate (containing 15% ascorbic acid, pH 5.0) at 37°C for 0.5 h. Finally, the absorbance of the above mixture was detected at 650 nm and the P content was normalized by fresh weight (Zhu et al., 2018).

### Measurement of Total P Content

Rice seedlings were first rinsed with distilled water, then separated into roots and shoots, and weighed. After the samples were dried in an oven at 75°C for 3 days, 4 ml of HNO_3_ was added. The above mixture was incubated at 130°C for 24 h, and after cooling, ultrapure water was added to reach a final volume of 20 ml. The P concentration in the solution was measured by inductively coupled plasma atomic emission spectroscopy (ICP-AES; Fisons ARL Accuris, Ecublens, Switzerland).

### Measurement of the P Concentration in Xylem Sap

Rice seedlings were first excised with a razor 2 cm above the root, and then the xylem sap was collected (Che et al., 2016). Two hours later, the volume of the xylem sap was recorded, and 1 ml of distilled water was added for the measurement of P content by ICP-AES.

### Extraction of Cell Wall and Cell Wall Pectin

For cell wall extraction, roots were first ground in liquid nitrogen and 8 ml of 75% ethanol was added. After incubation at 4°C for 20 min, samples were centrifuged at 12,000*g* for 20 min and the pellet was collected. Next, 8 ml of acetone was added to the pellet, and after a 20 min incubation, the supernatant was removed as described above and the pellet was extracted with 8 ml of chloroform:methyl alcohol (1:1) and again with 8 ml of methyl alcohol. Finally, the pellet, containing cell wall material, was dried in a freeze dryer.

Pectin was extracted as follows: 1 ml of 100°C ultrapure water was added to about 5 mg of the extracted cell wall and incubated for 1 h. Next, the mixture was centrifuged at 12,000*g* for 8 min and the supernatant was collected. The pellet was extracted with 1 ml ultrapure water two more times and the supernatants were collected as described above. The collected supernatants were regarded as pectin solution (Zhong and Lauchli, 1993).

### Measurement of the Uronic Acid Content in Pectin

A 200 μl aliquot of the extracted pectin solution was transferred to a 2 ml tube, then 1 ml of 98% H_2_SO_4_ (consisting of 12.5 mM Na_2_B_4_O_7_·10H_2_O) was added. After incubation in a 100°C water bath for 5 min, samples were chilled, and 20 μl of 0.15% M-hydro-diphenyl (m:v) was added. Finally, the mixture was incubated at 25°C for 20 min and the absorbance was measured at 520 nm (Blumenkrantz and Asboe-Hansen, 1973). Galacturonic acid (Sigma) was used as a standard.

### Measurement of the Pectin Methylesterase Activity

About 5 mg of cell wall materials were weighed, and then 1 ml of 1 M NaCl (dissolved in 10 mM Tris buffer, pH 6.0) was added to extract the PME enzyme at 4°C. Then, the mixture was centrifuged at 12,000*g* for 15 min and the supernatant was collected. Next, 0.1 ml of 200 mM PBS solution (containing 640 μg ml^−1^ pectin; pH 7.5) and 10 μl of alcohol oxidase were added and incubated at 30°C for 10 min. Then, 0.2 ml of 0.5 M NaOH (containing 5 mg ml^−1^ purpald) was added and the mixture was incubated at 30°C for 30 min. Finally, the absorbance was measured at 550 nm (Zhu et al., 2016).

### Measurement of the Cell Wall P Content

To measure the cell wall P content, 1 ml of 2 M HCl was added to 5 mg of cell wall extract and allowed to sit for 24 h with occasional shaking. The mixture was then centrifuged at 13,000*g* for 5 min and the supernatant was collected, and ultrapure water was added to bring to a final volume of 5 ml. The P content was measured by ICP-AES.

### Measurement of Ethylene Production

For determination of ethylene production, shoots were collected after the treatments and placed into 8 ml glass vials and rapidly sealed as reported by Wu et al. (2011). Then, shoot samples were incubated at 25°C in the dark for 4 h. Gas (1 ml) was withdrawn from the airspace of each vial using a gas-tight syringe (Focus GC, Thermo, USA) and injected into a gas chromatograph (Focus GC, Thermo, USA) equipped with a capillary column (CP-carboPLOT P7, Varian, CA, USA) and flame-ionization detector.

### Measurement of the Photosynthesis Rate

The net photosynthesis rate of the youngest fully expanded leaf was measured with a LI-6400 Infrared Gas Analyzer (Li-Cor Biosciences) using a red-blue light at a PAR intensity of 1,200 μmol photons m^−2^ s^−1^, a constant CO_2_ concentration of 400 μl L^−1^, a 25°C leaf temperature, and 40% relative humidity (Sun et al., 2018).

### Gene Expression Analysis

After the treatments, roots were collected and instantly frozen in liquid nitrogen. The extraction of total RNA, the conversion of the total RNA to cDNA, and the subsequent quantitative PCR were all conducted according to Zhu et al. (2016). The PCR reaction mixture consisted of RNase-free water (2.6 μl), cDNA (2 μl), SYBR Premix (5 μl, Toyobo, Japan), forward primer (0.2 μl), and revise primer (0.2 μl). The primers are listed in Table 1 (Zhu et al., 2016). Three replications were used for each cDNA sample, and the relative expression of the candidate genes was calculated by the 2^-ΔΔCT^ method (Livak and Schmittgen, 2001). *OsHistone* was used as a reference gene (Xia et al., 2013).

**Table 1:** Gene-specific primers used in this work.

Gene	Forward (5′-3′)	Reverse (5′-3′)
*OsPT2*	GACGAGACCGCCCAAGAAG	TTTTCAGTCACTCACGTCGAGAC
*OsPT6*	TATAACTGATCGATCGAGACCAGAG	TGGATAGCCAGGCCAGTTATATATC
*OsPT8*	AGAAGGCAAAAGAAATGTGTGTTAAAT	AAAATGTATTCGTGCCAAATTGCT
*OsHistone H3*	GGTCAACTTGTTGATTCCCCTCT	AACCGCAAAATCCAAAGAACG

### Statistical Analysis

Three replications were used for each set of experiments. Data were analyzed by one-way analysis of variance (ANOVA) and the means were compared by Duncan’s multiple range test. Different letters on the histograms indicate that the means were statistically different at *p* < 0.05.

## Results

### Elevated CO_2_ Improves the Growth of Rice Seedlings

**Figure 1:**
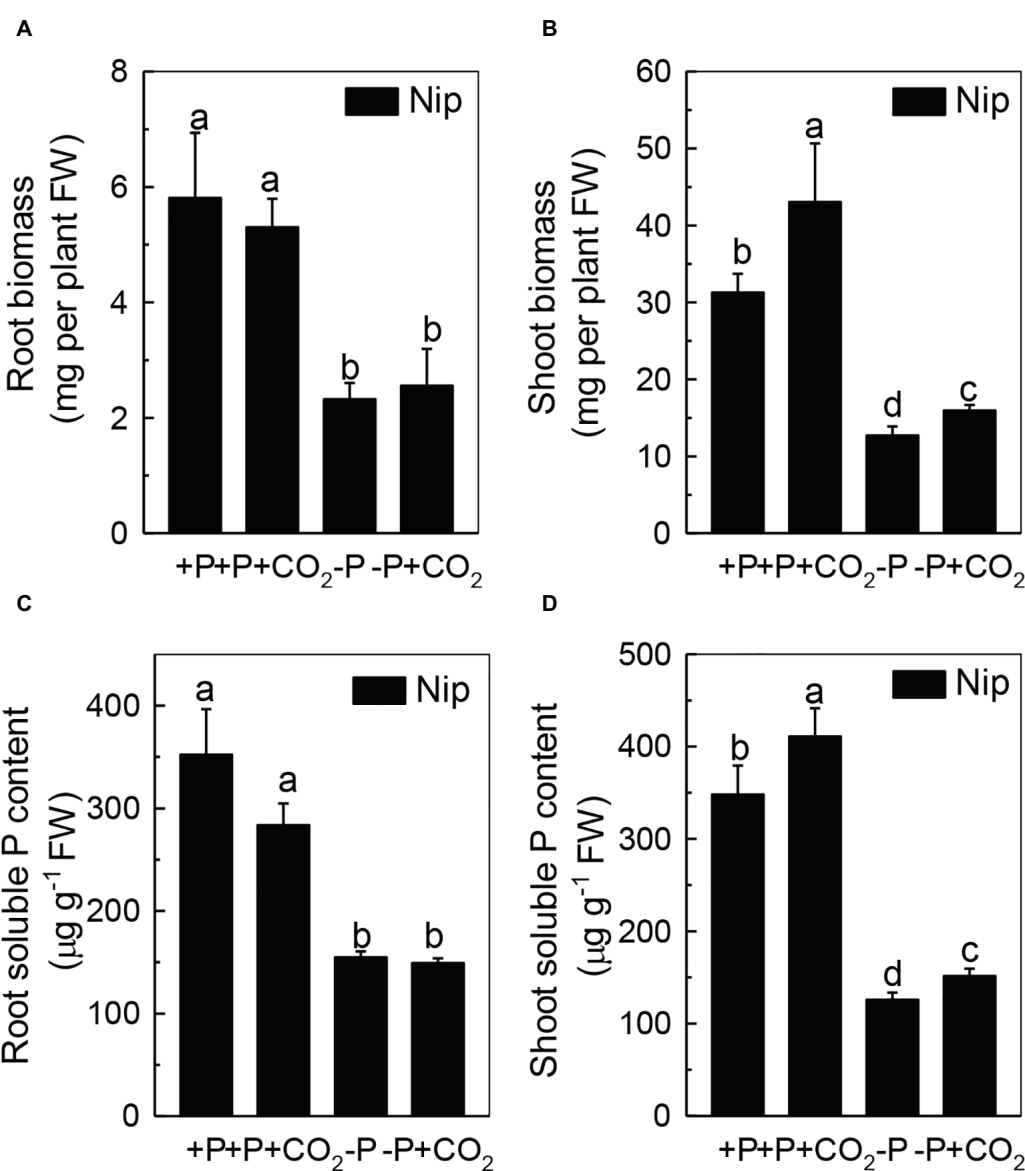
Effects of elevated CO_2_ on the biomass of rice roots and shoots **(A,B)** and the soluble P content in rice roots and shoots **(C,D)** grown under +P and −P conditions for 1 week. Data are means ± SD (*n* = 4). Columns with different letters are significantly different at *p* < 0.05.

To investigate the response of rice to P deficiency under elevated CO_2_, 2-week-old rice seedlings were grown in either a P-sufficient or P-deficient solution in ambient or elevated CO_2_ for 7 days. As shown in Figures 1A,B, P deficiency reduced the growth of roots and shoots; however, shoot growth was improved under elevated CO_2_, independent of the P status, when compared with ambient CO_2_, while root growth was nearly the same in both ambient and elevated CO_2_ treatments. These results indicated that elevated CO_2_ can improve shoot growth. Then, a question raised why elevated CO_2_ can improve the growth of shoots in both P-sufficient and P-deficient conditions. As CO_2_ is an important substrate molecule for photosynthesis, and higher concentrations of CO_2_ would affect the photosynthesis rate, we wondered whether elevated CO_2_ could alleviate the reduction of rice plant growth caused by P deficiency. As shown in Supplementary Figure 1, while P deficiency significantly decreased the photosynthesis rate, elevated CO_2_ significantly promoted the photosynthesis rate, indicating that the enhanced shoot growth observed under elevated CO_2_ may be related to an enhanced photosynthesis rate. Moreover, soluble P accumulated in the shoot under elevated CO_2_ (Figure 1D); however, no alteration was found in the root soluble P content (Figure 1C), indicating that elevated CO_2_ can stimulate more efficient reutilization of P in shoots. However, under P deficiency, no significant difference in the total P content was found between elevated CO_2_ and ambient CO_2_ conditions (Supplementary Figure 2); the underlying reason may be that the plants failed to take up external P under the −P conditions.

### Elevated CO_2_ Significantly Increased the Reutilization of Shoot Cell Wall P

**Figure 2:**
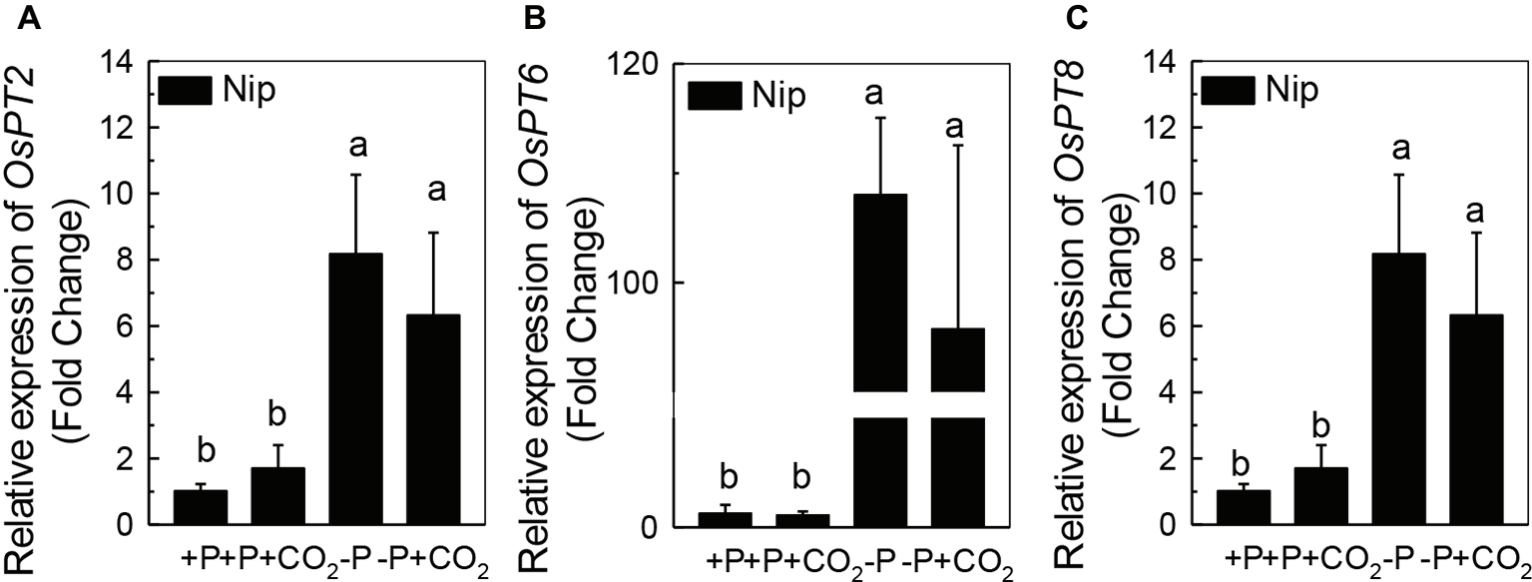
Effect of elevated CO_2_ on the expression of *OsPT2* **(A)**, *OsPT6* **(B)**, and *OsPT8* **(C)** in rice roots grown under +P and −P conditions for 1 week. Data are means ± SD (*n* = 4). Columns with different letters are significantly different at *p* < 0.05.

**Figure 3:**
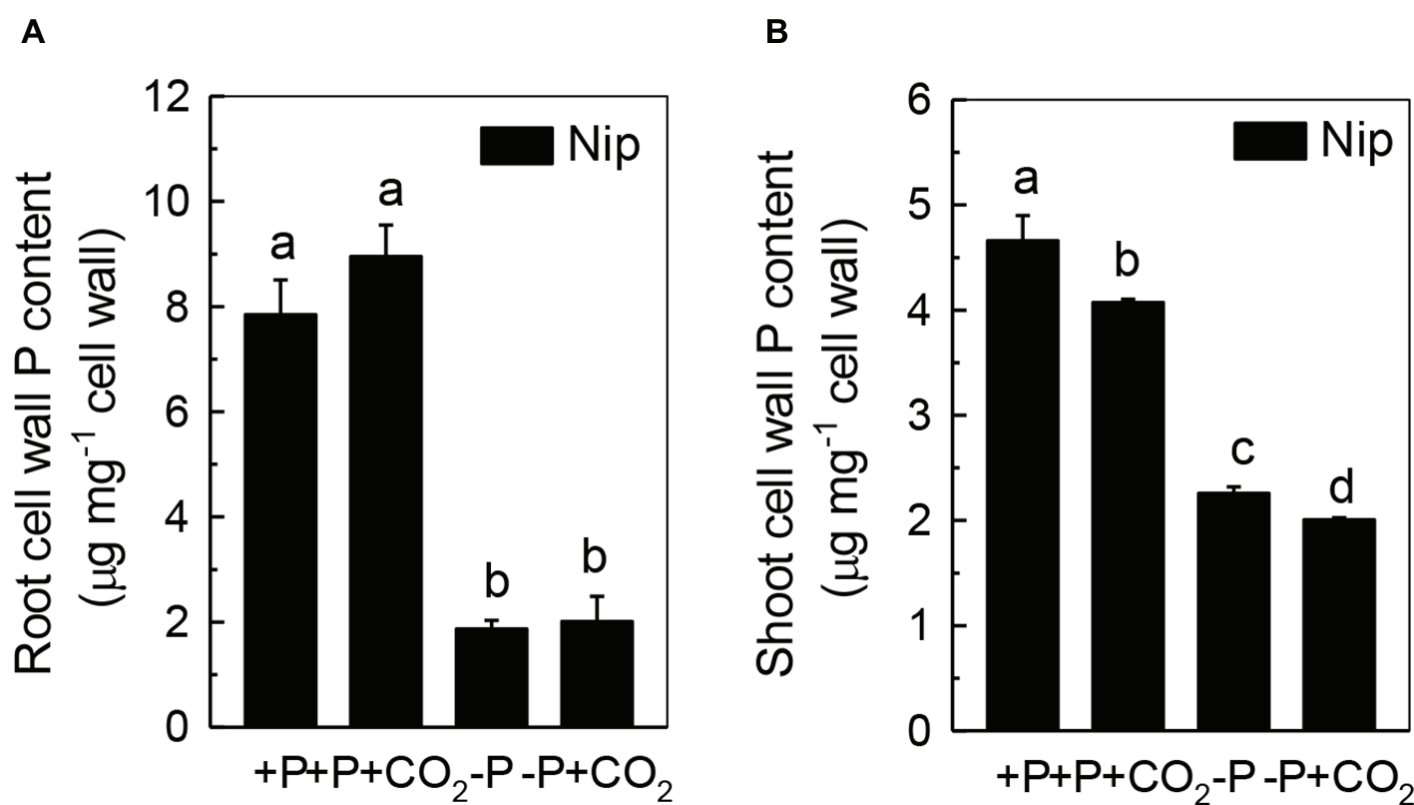
Effects of elevated CO_2_ on root cell wall P content **(A)** and shoot cell wall P content **(B)** in rice grown under +P and −P conditions for 1 week. Data are means ± SD (*n* = 44). Columns with different letters are significantly different at *p* < 0.05.

We questioned whether the P that accumulated in the shoot under elevated CO_2_ originated from elevated P translocation from the roots or from elevated P reutilization from the shoot cell wall, as the cell wall acts as a P pool. Thus, we analyzed the expression of three phosphate transporter genes (*OsPT2*, *OsPT6*, and *OsPT8*) that are responsible for root-to-shoot P translocation. As shown in Figure 2, P deficiency significantly induced the expression of *OsPT2*, *OsPT6*, and *OsPT8* under the ambient CO_2_ condition (−P), while elevated CO_2_ (-P + CO_2_) had no further effect, which was also observed in the P-sufficient conditions, indicating that root-to-shoot P translocation may not be involved in the elevated soluble P content observed in shoots under elevated CO_2_. To clarify whether elevated CO_2_ stimulates root-to-shoot P translocation, we also measured the P content in xylem sap. Although significantly less P was observed in xylem sap when rice was grown under the P-deficient conditions, elevated CO_2_ had no further effect (Supplementary Figure 3). In addition, we found no alteration of the root cell wall P content (Figure 3A), while the P content in the shoot cell wall was decreased under elevated CO_2_ (Figure 3B). These results further suggested that the elevated soluble P content observed in shoots under elevated CO_2_ is attributed to the reutilization of shoot cell wall P.

### Elevated CO_2_ Significantly Increased the Shoot Cell Wall Pectin Content

**Figure 4:**
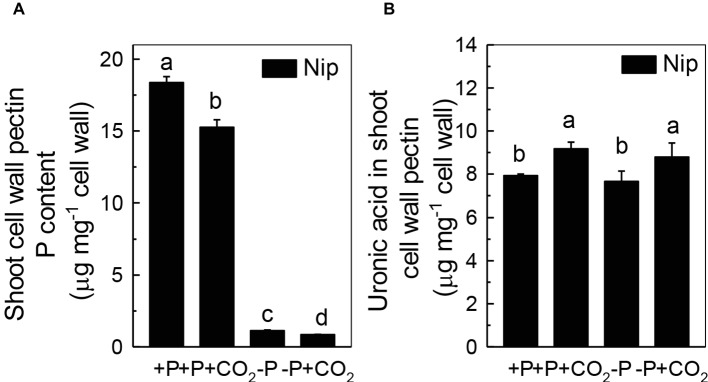
Effects of elevated CO_2_ on P content **(A)** and uronic acid content in shoot cell wall pectin **(B)** in rice grown under +P and −P conditions for 1 week. Data are means ± SD (*n* = 4). Columns with different letters are significantly different at *p* < 0.05.

Previous studies demonstrated that pectin contributes greatly to cell wall P reutilization in rice (Zhu et al., 2015); thus, we examined the role of pectin in rice under P-deficiency and elevated CO_2_ conditions. As expected, less P was adsorbed in shoot cell wall pectin under elevated CO_2_ compared to ambient CO_2_, indicating a greater P-release potential in the pectin fraction under elevated CO_2_ (Figure 4A). In agreement with this, we found an increase of the pectin content under elevated CO_2_, irrespective of the P status (Figure 4B). However, considering that about 88% of pectin-released P accounts for the net soluble P gained in the shoot under the −P condition (As shown in Figure 4A, the shoot cell wall pectin-retained P was decreased from 1.13 μg mg^−1^ cell wall in the −P treatment to 0.85 μg mg^−1^ cell wall in the −P + CO_2_ treatment, while as shown in Figure 1D, the shoot soluble P was increased from 125.9 μg g^−1^ fresh weight in the −P treatment to 151.4 μg g^−1^ fresh weight in the −P + CO_2_ treatment. As 1 g of fresh shoot can provide about 80 mg cell wall, thus about 22.4 μg P was released from 80 mg Nip cell wall pectin in the −P + CO_2_ treatment when compared with −P treatment alone, which accounts for 88% of the net soluble P gained – 25.5 μg g^−1^ fresh weight in the −P + CO_2_ treatment when compared with −P treatment. The almost complete absence of any change in the cell wall pectin content and its P retention in rice roots under both ambient and elevated CO_2_ conditions, this again excludes the involvement of the roots in the increase of P reutilization under elevated CO_2_ (Supplementary Figure 4), Thus, we focused on shoots instead of roots in the following examinations.

### Elevated CO_2_ Significantly Increased the Pectin Methylesterase Activity in the Shoot

**Figure 5:**
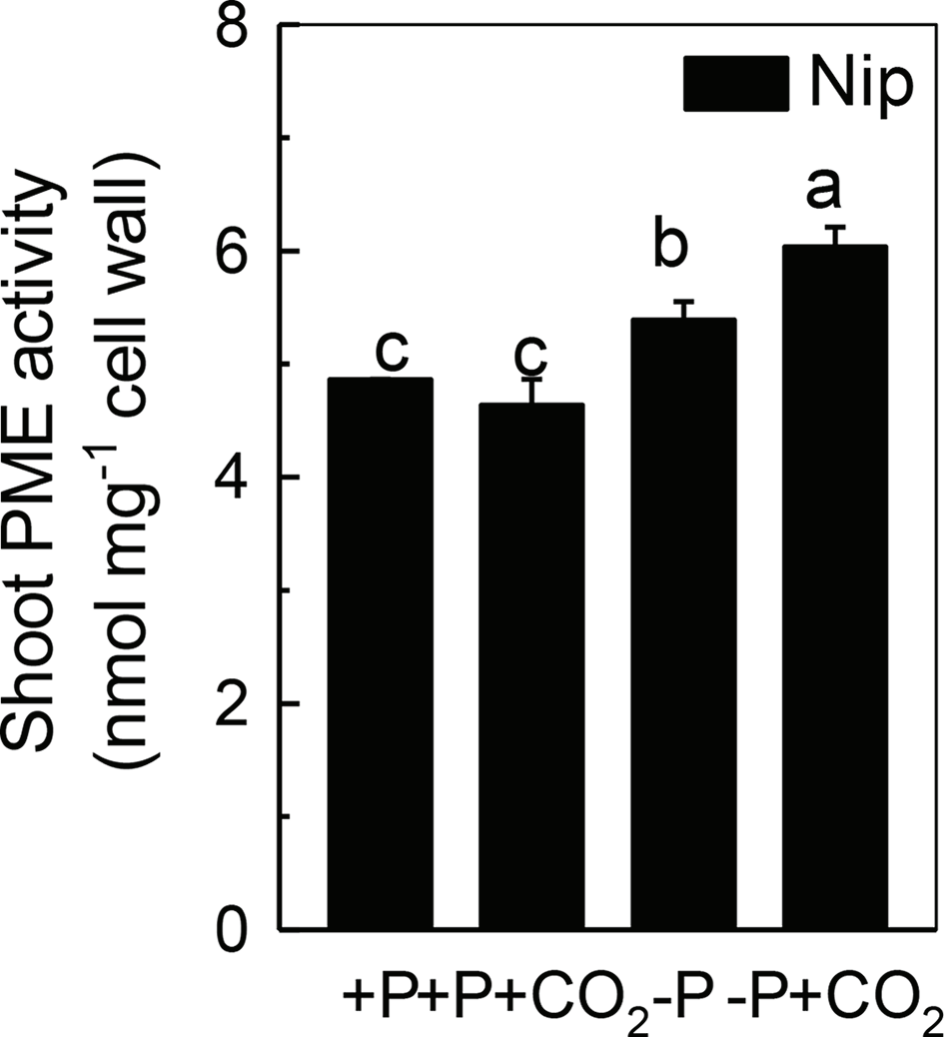
Effects of elevated CO_2_ on PME activity in shoot cell wall in rice grown under +P and −P conditions for 1 week. Data are means ± SD (*n* = 4). Columns with different letters are significantly different at *p* < 0.05.

Only de-esterified pectin will present a net negative charge in the cell wall space, and this negative charge is generated from the demethylation of pectin, a process that controlled by PME. Thus, we measured the PME activity in the shoot cell wall. As shown in Figure 5, P deficiency significantly induced the PME activity in the shoot cell wall, and this increment was further amplified under elevated CO_2_, indicating that the negative charges generated by PME are also involved in the increased reutilization of shoot cell wall P under elevated CO_2_.

### Elevated CO_2_ Significantly Increased Ethylene Production in Rice Shoots

**Figure 6:**
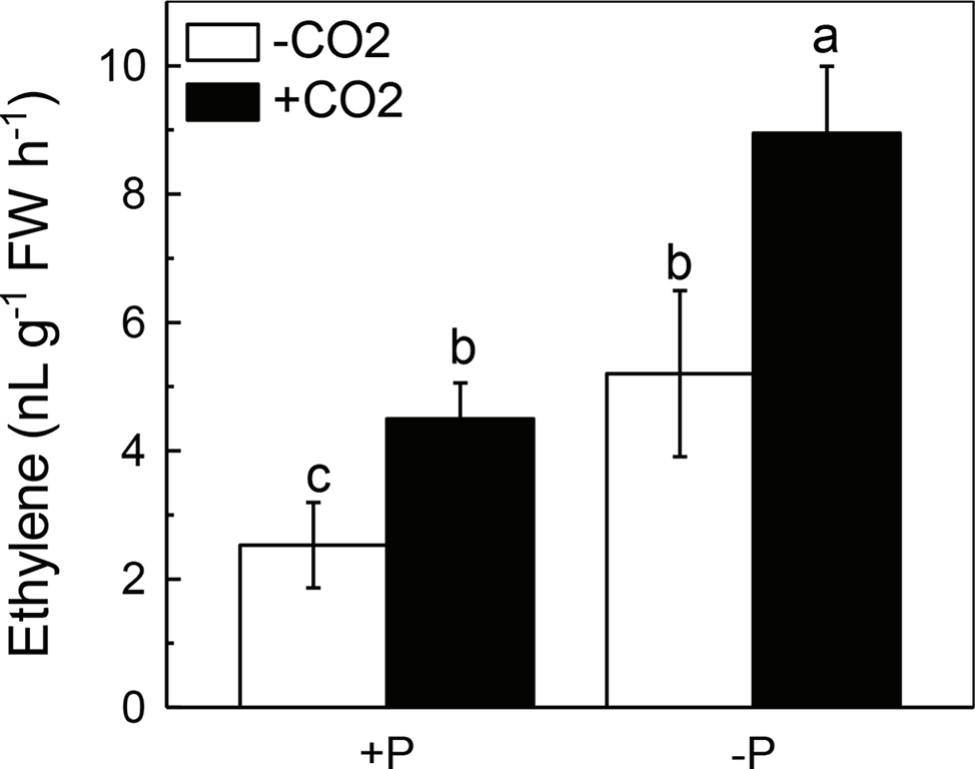
Effects of elevated CO_2_ on ethylene production in rice shoots grown under +P and −P conditions for 1 week. Data are means ± SD (*n* = 4). Columns with different letters are significantly different at *p* < 0.05.

**Figure 7:**
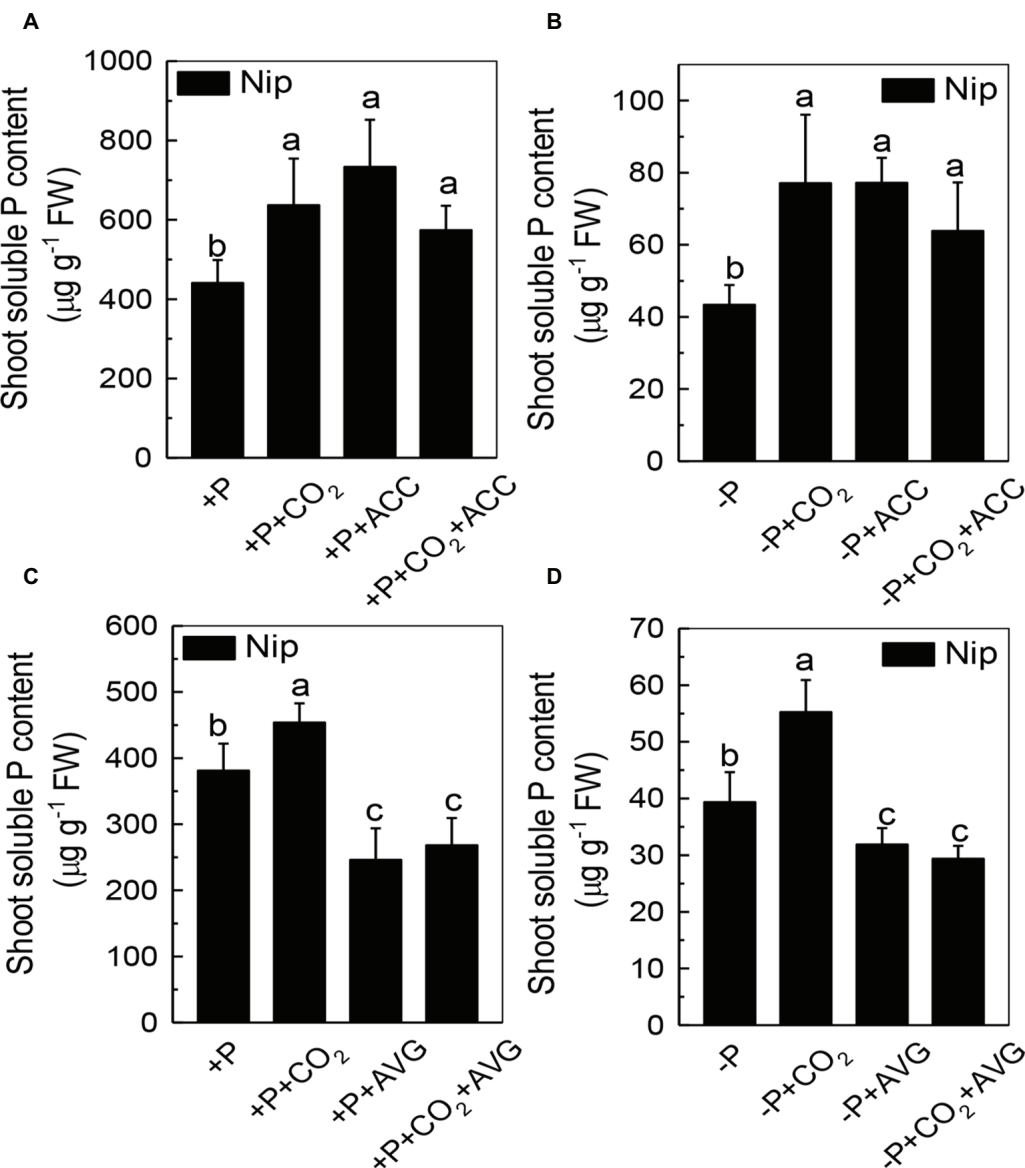
Effects of different treatments on the soluble P content in rice roots **(A,C)** and shoots **(B,D)** grown under +P and −P conditions for 1 week. Data are means ± SD (*n* = 4). Columns with different letters are significantly different at *p* < 0.05.

We next questioned how elevated CO_2_ can increase the reutilization of shoot cell wall P. As ethylene is a signal that is closely related to the P reutilization in the rice cell wall (Zhu et al., 2016, 2017), we hypothesized that there could be a relationship between elevated CO_2_ and ethylene. As expected, P deficiency significantly induced ethylene production in rice roots, and elevated CO_2_ further induced this increment of ethylene production (Figure 6). To determine whether this induction of ethylene production is involved in the accelerated P reutilization observed under elevated CO_2_, we applied a precursor of ethylene (ACC) and an inhibitor of ethylene (AVG). As shown in Figure 7, the ACC treatment had a similar effect as that of the elevated CO_2_ treatments in that we found more shoot soluble P under the ACC treatment irrespective of the P status, while the results were opposite for the AVG treatment, which further confirmed the role of ethylene in the increased reutilization of shoot cell wall P under elevated CO_2_.

## Discussion

Elevated CO_2_ can affect plant growth by increasing the requirements for macronutrients, such as P and nitrogen (N; Kogawara et al., 2006; Lagomarsino et al., 2008), and also by improving P nutrition (Niu et al., 2013); facilitating the reutilization of iron (Fe; Jin et al., 2009); and alleviating Al toxicity in rice (Zhu et al., 2017). However, how elevated CO_2_ facilitates the reutilization of P in rice remains elusive. In the present study, we found that elevated CO_2_ can stimulate more efficient reutilization of shoot cell wall P (Figure 1), as well as increase the pectin content and PME activity of the shoot cell wall (Figures 4, 5), which in turn releases cell wall P (Figure 3), resulting in more soluble P in the rice shoot (Figure 1). Furthermore, ethylene appears to participate in this process (Figures 6, 7). To our knowledge, this is the first study to show that elevated CO_2_ can affect the reutilization of shoot cell wall P in rice.

As the P in cell walls accounts for approximately 50% of the root total P in rice (Zhu et al., 2015), any approaches to improve the reutilization of the P from the cell wall may promote plant growth under P-deficient conditions in rice. Studies in rice have shown a high correlation of the cell wall-released P and the root/shoot soluble P content (Zhu et al., 2015, 2016, 2018). Moreover, Yu et al. (2016) recently identified one Arabidopsis mutant, which lacks the function of *AMMONIUM-OVERLY-SENSITIVE1*, *amos1*, that released more P from its root cell wall under P-deficient conditions and thus was highly resistant to P deficiency. In this study, elevated CO_2_ significantly increased the release of P from the rice shoot cell wall (Figure 3), which in turn increased the soluble P content in rice shoots (Figure 1), indicating a cell wall P-based P reutilization mechanism. Furthermore, as the pectin content is the major factor that positively determines the efficiency of the release of cell wall P under P deficiency (Zhu et al., 2015), we investigated whether pectin contributed to the increased reutilization of cell wall P. As expected, elevated CO_2_ significantly increased the pectin content and decreased P retention within this polysaccharide under the −P condition (Figure 4), indicating that pectin contributes to the increased P reutilization in rice shoot cell walls under elevated CO_2_.

Pectin is a major polysaccharide in the cell wall and consists of rhamnogalacturonan I (RG I), rhamnogalacturonan II (RG II), galactans, arabinans, homogalacturonan (HGA), and other polysaccharides (Cosgrove, 2005). Several studies have indicated that the negatively charged carboxylic groups in the D-galacturonic acid component of the pectic network of plant cell walls have a high affinity for Al^3+^ and Fe^3+^; thus, this network possesses the ability to solubilize Al-bound P and Fe-bound P in the cell wall, and as a result, the insoluble P can be reutilized (Ae et al., 1996; Gessa et al., 1997). Zhu et al. (2015) demonstrated that pectin participates in the cell wall P reutilization mechanism by comparing the pectin content and root soluble P content in 14 different rice cultivars cultured in −P nutrient solution for 1 week. In addition, homogalacturonans, the main component of pectin, are first synthesized in the Golgi apparatus in the methyl esterified form. After secretion into the cell wall, the demethylation process of pectin occurs (the removal of -CH_3_- from carboxyl groups), which is catalyzed by PME (Ostatek and Blamey, 1995; Ridley et al., 2001). This results in high amounts of negatively charged groups, and as a result, the binding capacity to cations is enhanced (Cosgrove, 2005; Willats et al., 2006). Therefore, the higher the PME activity, the more negatively charged groups in pectin, and more P is released from the cell wall. As expected, in the present study, elevated CO_2_ significantly increased the PME activity in the P-deficient rice shoots (Figure 5), indicating that elevated CO_2_ enhances the remobilization of cell wall P in rice shoots by stimulating pectin biosynthesis and increasing PME activity.

Roots act as a P pool for the shoots. Once P is released from the cell wall to the symplasm, three phosphate transporters, OsPT2, OsPT6, and OsPT8, are responsible for the translocation of P to the shoots *via* symplasmic transport to the xylem parenchyma cells, followed by secretion to the xylem (Jeschke et al., 1997; Ai et al., 2009; Jia et al., 2011). It is interesting that although P deficiency significantly induced the expression of the genes encoding these phosphate transporters, elevated CO_2_ had no further effect (Figure 2). The none alteration of the xylem P content between −P and −P + CO_2_ treatment further indicated that the increased soluble P found in the shoot was not derived from the root (Supplementary Figure 3).

Then, how does elevated CO_2_ increase the shoot cell wall pectin content and then increase P reutilization? Ethylene has been demonstrated to be a key molecule involved in P deficiency, such as facilitating P reutilization in rice (Zhu et al., 2016, 2017). Here, elevated CO_2_ significantly increased the P deficiency-induced production of ethylene in the shoots (Figure 6), and the addition of the ethylene precursor ACC and the inhibitor of ethylene (AVG) made no difference of the shoot soluble P content between the ambient and elevated CO_2_ conditions (Figure 7), indicating that the effect of elevated CO_2_ on rice under P deficiency may be related to the increased production of ethylene.

In conclusion, we have demonstrated that elevated CO_2_ can increase the reutilization of shoot P in rice. This reutilization is mediated by a cell wall P-based P remobilization mechanism involving an increase of pectin content and PME activity. Ethylene may act downstream of CO_2_ to control these processes; however, this putative mechanism needs further clarification.

## Author Contributions

XFZ, XLZ, and XYD performed the research. XFZ analyzed the data and wrote the draft. XFZ and RFS designed the research. RFS wrote the article.

### Conflict of Interest Statement

The authors declare that the research was conducted in the absence of any commercial or financial relationships that could be construed as a potential conflict of interest.

## Supplementary Material

SUPPLEMENTARY FIGURE 1 Effects of elevated CO_2_ on the photosynthesis rate in rice grown under +P and −P conditions for 1 week. Data are means ± SD (*n* = 4). Columns with different letters are significantly different at *p* < 0.05.

SUPPLEMENTARY FIGURE 2 Effects of elevated CO_2_ on total root **(A)** and shoot **(B)** P content in rice grown under +P and −P conditions for 1 week. Data are means ± SD (*n* = 4). Columns with different letters are significantly different at *p* < 0.05.

SUPPLEMENTARY FIGURE 3 Effects of elevated CO_2_ on the P content in the xylem sap of rice grown under +P and −P conditions for 1 week. Data are means ± SD (*n* = 4). Columns with different letters are significantly different at *p* < 0.05.

SUPPLEMENTARY FIGURE 4 Effects of elevated CO_2_ on the P content **(A)** and uronic acid content in root cell wall pectin **(B)** in rice grown under +P and −P conditions for 1 week. Data are means ± SD (*n* = 4). Columns with different letters are significantly different at *p* < 0.05.
