# Supplementary material for: Carbon Dioxide Improves Phosphorus Nutrition by Facilitating the Remobilization of Phosphorus From the Shoot Cell Wall in Rice (Oryza sativa)
Source: Front Plant Sci. 2019 May 22;10:665. doi: 10.3389/fpls.2019.00665 (PMC6541036; doi:10.3389/fpls.2019.00665)
Supplement: SUPPLEMENTARY FIGURE 1 — Effects of elevated CO2 on the photosynthesis rate in rice grown under +P and −P conditions for 1 week. Data are means ± SD (n = 4). Columns with different letters are significantly different at p < 0.05. [file Table_1.DOCX]

**Supplemental Fig. 1**

**Supplemental Fig. 2**

**Supplemental Fig. 3**

**Supplemental Fig. 4**
